# *Vaccinia Virus* Converts Microglia into Potent Oncolytic Agent for Glioblastoma and Neuroblastoma

**DOI:** 10.3390/cells14241943

**Published:** 2025-12-08

**Authors:** Elena Ekrami, Parisa Ghanbari, Eman M. Othman, Aladar A. Szalay

**Affiliations:** 1 Cancer Therapy Research Center (CTRC), Department of Biochemistry-I, Biocenter, University of Würzburg, 97074 Würzburg, Germany; elena.ekrami@uni-wuerzburg.de (E.E.); parisa.ghanbari@stud-mail.uni-wuerzburg.de (P.G.); 2 Department of Pathology, Center for Clinical Science Research, Stanford School of Medicine, Stanford, CA 94305, USA; 3 Department of Radiation Oncology, Rebecca & John Moores Comprehensive Cancer Center, University of California, San Diego, CA 92093, USA

**Keywords:** oncolytic virotherapy, *vaccinia virus*, microglia, glioblastoma, neuroblastoma

## Abstract

**Highlights:**

**What are the main findings?**

- Microglia infected with *vaccinia virus* (VV) efficiently deliver viruses to neuroblastoma and glioblastoma cells.
- Infected microglia promote oncolysis and viral spread in both 2D and 3D tumor models.

**What are the implications of the main findings?**

- Microglia act as effective carriers for VV.
- This platform can enhance oncolytic virotherapy and potentially integrate with immuno- or radiotherapy.

**Abstract:**

Background: Glioblastoma and neuroblastoma are aggressive brain and pediatric tumors with limited treatment options. *Vaccinia virus* (VV) has demonstrated potent oncolytic and immunomodulatory properties but faces challenges in tumor targeting. Methods: We developed an in vitro co-culture model using mouse microglia (BV2) infected with VV (LIVP strain) to deliver the virus to glioblastoma (U87) and neuroblastoma (SH-SY5Y) cells in both 2D and 3D systems. Our results demonstrated that VV-infected Microglia can efficiently deliver the virus to tumor cells, inducing substantial cytotoxic effects in both 2D and 3D models. Notably, the 3D cultures, which more closely mimic the native tumor microenvironment, exhibited the most pronounced enhancement in oncolysis. Conclusions: This microglia-based delivery strategy enables potent oncolytic activity against brain tumor cells and warrants further exploration in in vivo and combinational therapies.

## 1. Introduction

Oncolytic viruses (OVs) are a promising emerging class of anticancer immunotherapies that utilize the natural ability of specific replication-competent viruses to infect and selectively destroy tumor cells, while not harming non-neoplastic cells. Oncolytic viruses can be chosen from natural viral species due to their natural capacity to elicit immunogenic cell death (ICD) in cancerous cells; however, they may also be genetically modified to increase tumor selectivity, enhance replication competence, limit pathogenicity, and elevate immunogenicity.

OVs are a class of viruses capable of selectively attacking and destroying malignant cells within the tumor microenvironment, thereby slowing or halting tumor progression [1]. These viruses, whether naturally occurring or genetically engineered, replicate preferentially in cancer cells and exert direct cytotoxic effects. Unlike gene therapy, which uses viral vectors solely as delivery vehicles for therapeutic genes, OV therapy harnesses the virus itself as the active therapeutic agent [2]. Several OVs have been developed from diverse viral backbones, including *vaccinia virus*, *herpes simplex virus*, and *adenovirus*, each with unique properties and therapeutic advantages [1].

VV, a member of the *Poxviridae* family, has attracted particular interest in oncolytic virotherapy owing to its well-established safety record, hundreds of millions of individuwere immunized with various VV strains during the Smallpox Eradication Program (1950–1980) [2]. It replicates rapidly in the cytoplasm, initiates robust viral production within hours, and efficiently lyses a broad range of tumor cells owing to its diverse repertoire of entry and host-modulating proteins [3,4]. Depending on the tumor cell type, most infected cells undergo lysis within 48–72 h.

Challenges in VV therapy remain, particularly in delivering the virus efficiently to tumor sites. Intravenously administered VV must navigate multiple host immune barriers, including complement proteins, coagulation factors, blood cell components, and pre-existing neutralizing antibodies (nAbs), to target the tumor mass effectively [5,6].

To improve delivery and therapeutic efficacy, VV is increasingly combined with other anticancer treatments or administered using cellular carriers that can protect the virus from immune clearance and enhance its accumulation within tumors [7].

Neuroblastoma, the most common solid tumor of infancy, accounts for ~15% of childhood cancer deaths and typically requires intensive multimodal treatment [8,9]. Current treatment regimens typically span 18 months and may include chemotherapy, surgical resection, high-dose chemotherapy with autologous stem cell rescue, radiotherapy, immunotherapy, and isotretinoin.

Glioblastoma multiforme (GBM), the most common malignant brain tumor in adults, is a highly aggressive astrocytic tumor with a median survival of only 12–15 months despite multimodal therapy [10]. Standard therapies include surgery, concurrent radiation, and chemotherapy. However, the tumor’s infiltrative nature, frequent presence in eloquent brain regions, and propensity for recurrence make total surgical excision unlikely [11], accordingly; both Neuroblastoma and Glioblastoma cancers urgently require novel therapeutic strategies.

Microglia, the resident immune cells of the central nervous system, are actively recruited into the microenvironment of primary and metastatic brain tumors and respond to tumor-derived chemotactic signals by releasing cytokines, matrix metalloproteases, and growth factors [12]. Their robust tumor-homing capacity makes them an attractive platform for targeted delivery of oncolytic viruses.

In this study, we investigate a microglia-mediated delivery approach for oncolytic *vaccinia virus*. By infecting microglia with VV and co-culturing them with neuroblastoma and glioblastoma cells in both 2D and 3D models, we aim to use their natural migratory properties to enhance viral delivery and oncolytic effectiveness.

## 2. Materials and Methods

### 2.1. Cell Lines and Media

African green monkey kidney fibroblasts (CV-1), U87-MG glioblastoma cells, and BV2 murine microglial cells were obtained from the American Type Culture Collection (ATCC, Manassas, VA, USA). The human neuroblastoma cell line SH-SY5Y was kindly provided by Dr. Ignazio Caruana (University Hospital, Würzburg, Germany). CV-1, U87-MG, and SH-SY5Y cells were cultured in Dulbecco’s Modified Eagle Medium (DMEM; Gibco, Waltham, MA, USA) supplemented with 10% fetal bovine serum (FBS; Merck, Darmstadt, Germany) and 1% penicillin–streptomycin (Thermo Fisher Scientific, Waltham, MA, USA) at 37 °C in a humidified atmosphere of 5% CO_2_.

BV2 cells, derived from raf/myc-immortalized neonatal murine microglia, were maintained in RPMI-1640 medium (Gibco, Waltham, MA, USA) with 10% FBS and 1% penicillin–streptomycin under the same culture conditions. As a semi-adherent cell line, BV2 cells grow in both suspension and adherent states.

### 2.2. Virus Strain

The *vaccinia virus* strain LIVP 1.1.1, a plaque-purified isolate from the wild-type LIVP (Lister strain, Institute of Viral Preparations, Moscow, Russia).

### 2.3. Viral Replication and Plaque Assay

BV2 cells were seeded in 24-well plates and allowed to adhere for 24 h before infection with LIVP 1.1.1 at multiplicities of infection (MOIs) of 0.1, 1, or 10. Virus adsorption was carried out for 2 h at 37 °C with gentle swirling every 30 min, after which the inoculum was replaced with fresh medium. Cultures were monitored for four days using an IncuCyte live-cell imaging system (Sartorius, Göttingen, Germany).

At 24-, 48-, 72-, and 96-h post-infection, cell pellets and supernatants were collected and stored at −80 °C. Viral titers were determined by plaque assay on confluent CV-1 cell monolayers. Briefly, samples underwent three freeze–thaw cycles, followed by sonication and serial dilution in DMEM containing 2% FBS. Two hundred microliters of each dilution were added to duplicate wells and incubated for 1 h at 37 °C with gentle swirling every 15 min. A 1.5% sodium carboxymethyl cellulose (CMC; Sigma-Aldrich, St. Louis, MO, USA) overlay was then applied, and plates were incubated for 40 h. Monolayers were stained with crystal violet, and plaques were counted to calculate titters in PFU/mL.

For co-culture experiments, BV2 cells infected at MOI 1 were mixed 1:1 with U87-MG or SH-SY5Y cells, and viral titers from supernatants and pellets were quantified at 24, 48, 72, and 96 h as described above.

### 2.4. MTT Cell Viability Assay

Cell viability was assessed by mitochondrial reduction of 3-(4,5-dimethylthiazol-2-yl)-2,5-diphenyltetrazolium bromide (MTT; Cell Proliferation Kit, Roche Diagnostics GmbH, Mannheim, Germany) to formazan. BV2 cells (1 × 10^4^/well) were seeded in 96-well plates, infected with LIVP 1.1.1 at MOIs of 0.1, 1, or 10, and incubated for 24–96 h. MTT solution was added to a final concentration of 0.5 mg/mL and incubated for 3–4 h until formazan crystals formed. Crystals were dissolved in isopropanol, and absorbance was read at 570 nm (reference 620 nm). Non-infected controls were set as 100% viability.

For tumor cell co-culture assays, U87-MG or SH-SY5Y cells (5 × 10^3^/well) were seeded and co-cultured 1:1 with either non-infected or VV-infected BV2 cells (MOI 1). Viability was quantified by MTT assay as above, with non-infected BV2/tumor co-cultures serving as controls.

### 2.5. Co-Culture Assays

BV2 cells (1 × 10^4^/mL) were seeded in 24-well plates and infected with LIVP 1.1.1 at MOI 1 for 48 h before co-culture. SH-SY5Y-GFP and U87-GFP cells were plated at 8 × 10^3^/well in 96-well plates 16 h prior to co-culture. Infected or non-infected BV2 cells were washed, resuspended in fresh medium, and added to tumor cells at ratios of 1:1 or 5:1. Cultures were imaged over time using the IncuCyte system.

### 2.6. Cell Invasion Assay

Invasion assays were performed using 24-well Matrigel-coated transwell chambers (8 μm pores; BD BioCoat, BD Biosciences, San Jose, CA, USA). U87-GFP or SH-SY5Y-GFP cells (5 × 10^4^) were seeded in the upper chamber. The lower chamber contained either non-infected BV2 cells, VV-infected BV2 cells (MOI 1), or DMEM with 10% FBS plus free VV. After 96 h, cells in the upper chamber were analyzed for invasion and infection status.

### 2.7. Scaffold-Free 3D Culture

BV2 cells were cultured in 24-well plates for 24 h, then infected with LIVP 1.1.1 at MOI 1 for 2 h with gentle swirling every 30 min. Infected and Non-infected BV2 cells were counted after 48 h.

SH-SY5Y-GFP and U87-GFP spheroids were generated in ultra-low attachment 24-well plates (Corning Inc., Corning, NY, USA) over 96 h. Infected or Non-infected BV2 cells (1 × 10^4^) were added to spheroids of equal tumor cell numbers. Spheroid growth and morphology were monitored daily for 10 days by IncuCyte imaging. After 48 h, spheroids were fixed in 4% formaldehyde, embedded in OCT medium (Tissue-Tek^®^, Sakura Finetek USA, Torrance, CA, USA), cryosectioned, and examined by fluorescence microscopy (Keyence Corporation, Osaka, Japan).

### 2.8. Statistical Analysis

Data are presented as mean ± standard error of the mean (SEM) from at least three independent experiments. For MTT and plaque assays, comparisons between two groups were made using Student’s *t*-test, with *p* ≤ 0.0001 considered significant. One-way ANOVA (Analysis of Variance) was used for multi-group comparisons in co-culture MTT and plaque assays, with *p* ≤ 0.005 considered significant. Statistical analyses were performed using GraphPad Prism (version 9).

## 3. Results

### 3.1. VV Efficiently Replicates in BV2 Microglia

**Figure 1:**
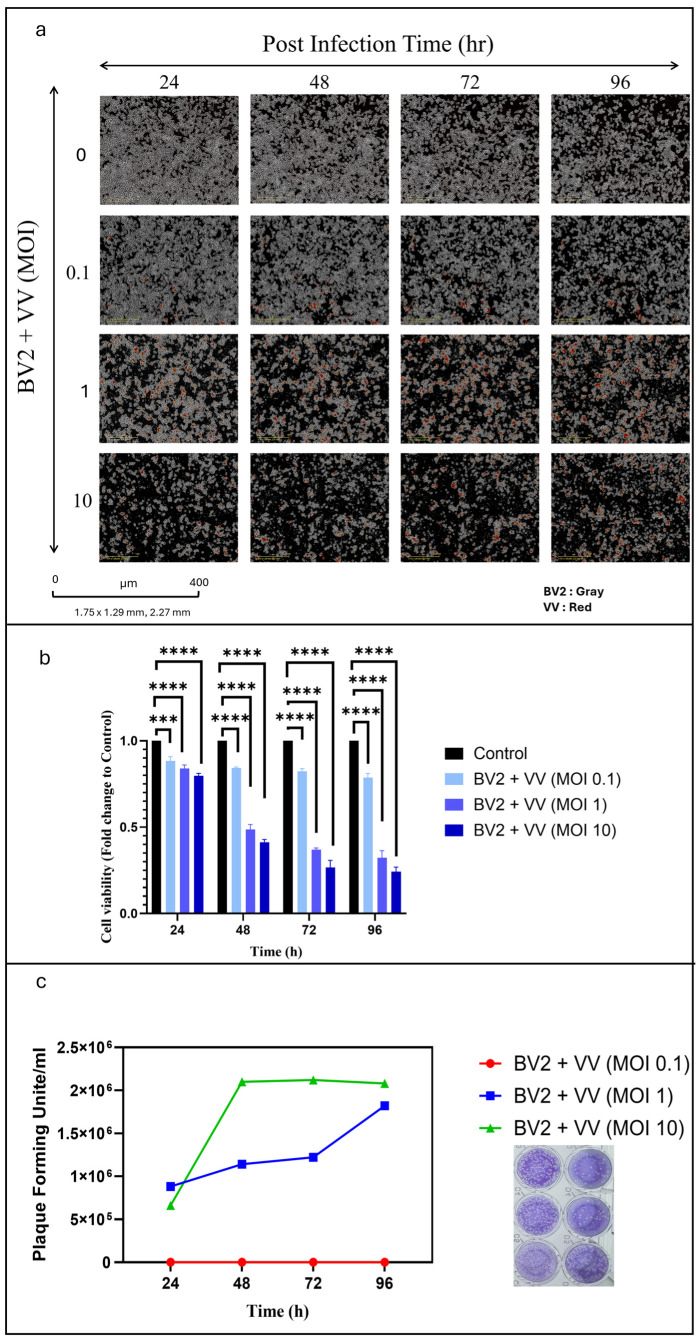
(**a**) IncuCyte images showing BV2 cell infection by VV at MOIs of 0.1, 1, and 10 (Red = *vaccinia virus*–infected Bv2 cells, Gray = BV2 microglia). (**b**) MTT assay results demonstrate that MOIs of 1 and 10 significantly reduced BV2 cell viability at 48 h post-infection. (**c**) Plaque assay showing time-dependent viral release from BV2 cells infected with VV. **** *p* < 0.0001; *** *p* < 0.005.

We first assessed the ability of VV to infect and replicate within BV2 cells. BV2 cultures were infected at different MOIs and monitored for four days using the IncuCyte live-cell imaging system. Time-lapse images confirmed efficient viral entry and replication at all MOIs, with peak infection observed within the first 48 h, as indicated by increasing red fluorescence intensity (Figure 1a). During this period, marked cell loss was evident at MOIs of 1 and 10.

The cytotoxicity of VV toward BV2 cells was quantified by MTT assay (Figure 1b). At MOI 0.1, cell viability remained comparable to Non-infected controls. In contrast, MOIs of 1 and 10 produced significant reductions in viability, with approximately 50% cell loss by 48 h.

To evaluate viral release, supernatants from infected BV2 cells were titrated by plaque assay on CV-1 monolayers. Following 1-h adsorption, CMC overlay, and 40-h incubation, plaques were visualized by crystal violet staining and counted (Figure 1c). While MOI 0.1 yielded minimal viral output, infections at MOIs of 1 and 10 produced substantially higher titers, confirming robust VV replication and release from BV2 cells over the course of infection.

### 3.2. VV-Infected Microglia Induce Cytotoxicity in Neuroblastoma and Glioblastoma Tumor Cell Lines

**Figure 2:**
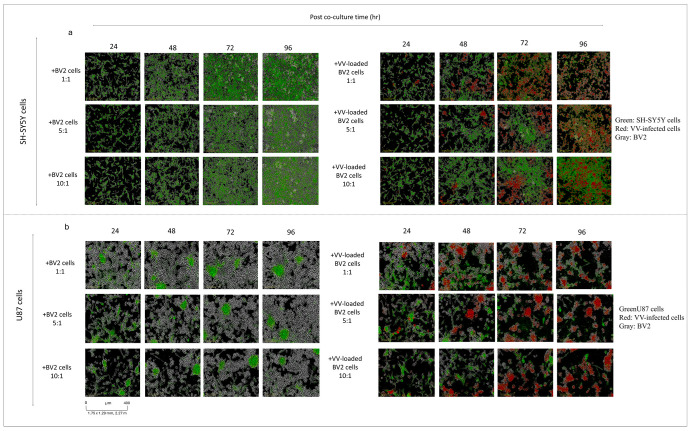
(**a**) IncuCyte images of SH-SY5Y cells co-cultured with VV-infected (MOI 1) or non-infected BV2 cells. (**b**) IncuCyte images of U87 cells co-cultured with VV-infected (MOI 1) or non-infected BV2 cells. (Green = GFP-expressing tumor cells (SH-SY5Y-GFP or U87-GFP), Red = *vaccinia virus*–infected cells, Gray = non-loaded BV2 Microglia cells).

BV2 cells, either Non-infected or infected with VV for 48 h, were co-cultured with SH-SY5Y-GFP neuroblastoma cells and U87-GFP glioblastoma cells. Cultures were monitored using the IncuCyte live-cell imaging system. Non-infected BV2 cells had no measurable effect on cancer cell survival or proliferation in either model (Figure 2). In contrast, VV-infected BV2 cells reduced tumor cell viability in a ratio-dependent manner. At 1:1 and 5:1 BV2-to-tumor cell ratios, SH-SY5Y-GFP cells were almost eliminated within 72 h (Figure 2a), while U87-GFP cells showed significant viability loss as early as 48 h post-coculture (Figure 2b).

### 3.3. Post-Coculture VV-Infected Microglia Enhance Viral Spread and Cancer Cell Death

**Figure 3:**
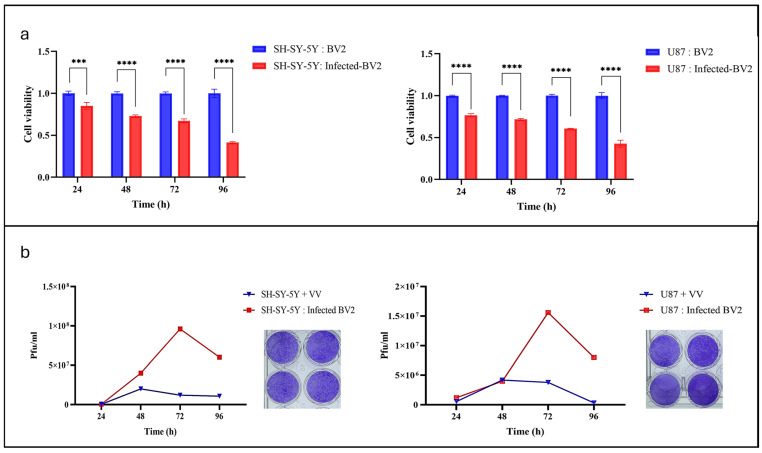
(**a**) MTT assay showing that VV-infected BV2 cells significantly reduced cancer cell viability at 72 h post-co-culture. (**b**) Plaque assay demonstrating that VV-infected BV2 cells co-cultured with cancer released infectious virus at multiple time points. *** *p* < 0.005; **** *p* < 0.0001.

Infected BV2 cells progressively reduced SH-SY5Y neuroblastoma viability compared with control co-cultures containing Non-infected BV2 cells, with viability dropping below 50% by day four. A similar pattern was observed in U87 glioblastoma co-cultures, confirming a consistent virus-mediated cytotoxic effect in both tumor models (Figure 3a).

Viral replication was quantified by plaque assay. Forty-eight hours post-infection, BV2 cells were co-cultured with SH-SY5Y or U87 cells, harvested, and overlaid on CV-1 monolayers for one hour before CMC overlay. After 40 h, plaques were visualized with crystal violet and counted (Figure 3b). Viral titers increased over time in both models, indicating active replication and spread within the co-culture environment. Notably, SH-SY5Y co-cultures produced higher viral loads than U87 co-cultures, suggesting that neuroblastoma cells may better support VV proliferation.

### 3.4. VV-Infected Microglia Suppress Tumor Cell Invasion and Promote Post-Coculture Viral Transfer

**Figure 4:**
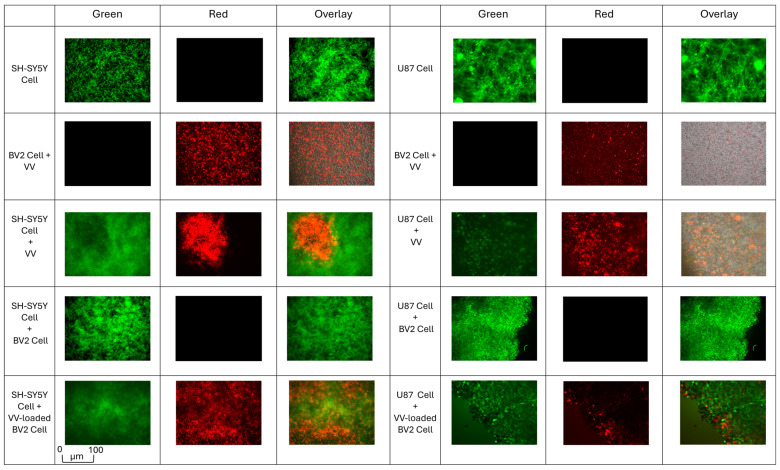
Fluorescence microscopy images of cancer cells in the upper chamber after 96 h, comparing conditions with non-infected BV2 cells, VV-infected BV2 cells, and 10% FBS with free VV. (Green = GFP-expressing tumor cells (SH-SY5Y-GFP or U87-GFP), Red = *vaccinia virus*–infected cells, Gray = non-loaded BV2 Microglia cells).

The objective of this experiment was to evaluate cancer cell migration and invasion using 24-well Matrigel-coated Transwell chambers over a 96-h incubation period. U87-GFP or SH-SY5Y-GFP cells were seeded into the upper chambers, while the lower chambers contained either non-infected BV2 cells, VV-infected BV2 cells (MOI 1), or DMEM supplemented with 10% FBS and free VV. After 96 h, the upper chamber was examined by fluorescence microscopy to assess both cancer cell invasion and infection status. As shown in Figure 4, non-infected BV2 cells had no effect on cancer cell growth in the upper chamber. In contrast, VV-infected BV2 cells migrated into the upper chamber and effectively suppressed cancer cell growth in neuroblastoma.

However, in glioblastoma, the cancer cells were infected but remained viable. Similarly, free VV alone was able to migrate to the upper chamber and suppress cancer cell proliferation.

### 3.5. VV-Infected Microglia Disrupt 3D Tumor Spheroid Structure and Cell Viability

**Figure 5:**
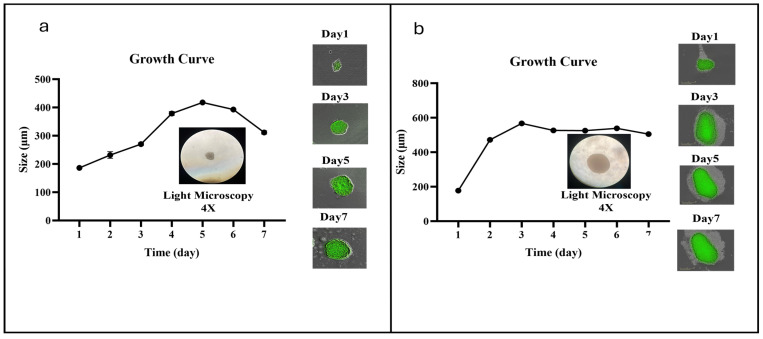
Growth kinetics of tumor spheroids. Images were captured daily using the IncuCyte system, and spheroid diameters were measured at 4× magnification. (**a**) Spheroid models SH-SY5Y are in growth phase from th 4th to 6th day, (**b**) Spheroid models of U87 cells are in the growth phase from the 2end to the 4th day.

Three-dimensional tumor spheroids were visualized by light microscopy at 4× magnification. Growth kinetics of SH-SY5Y monoculture spheroids were monitored over seven days, with spheroid size measured daily. As shown in Figure 5a, SH-SY5Y spheroids entered a pronounced growth phase between days 4 and 6; day 4 was selected as the optimal time point for subsequent experiments. Similarly, U87 monoculture spheroids exhibited peak growth between days 2 and 4 (Figure 5b), and day 3 was chosen for further analyses.

These findings demonstrate that VV-infected BV2 cells not only suppress tumor cell viability but also sustain robust viral amplification and release within the co-culture system, outperforming direct infection of cancer cells alone.

**Figure 6:**
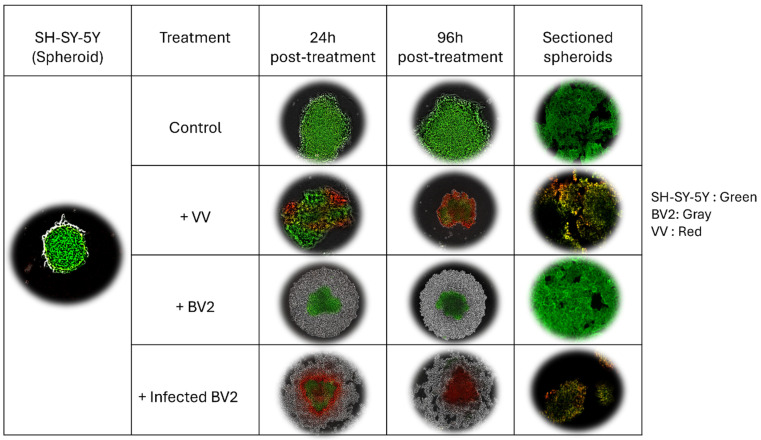
SH-SY5Y 3D spheroid models co-cultured with VV-infected or non-infected BV2 cells or directly treated with VV (MOI 1). Images were captured with the IncuCyte system; sectioned spheroids were visualized by fluorescence microscopy. (Green = GFP-expressing tumor cells (SH-SY5Y-GFP), Red = *vaccinia virus*–infected cells, Gray = non-loaded BV2 Microglia cells).

**Figure 7:**
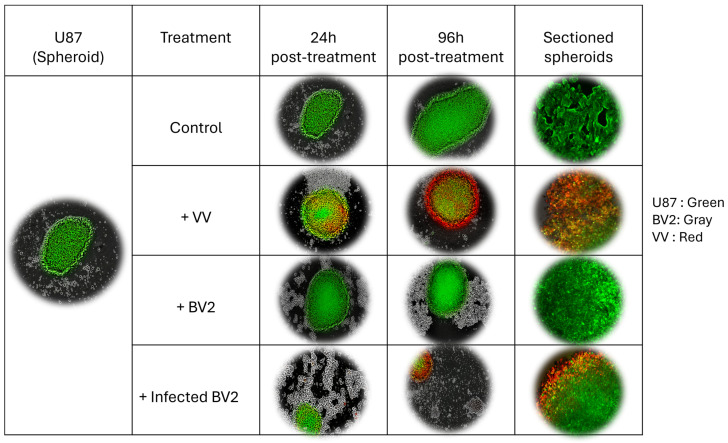
U87 3D spheroid models co-cultured with VV-infected or non-infected BV2 cells or directly treated with VV (MOI 1). Images were captured with the IncuCyte system; sectioned spheroids were visualized by fluorescence microscopy. (Green = GFP-expressing tumor cells (U87-GFP), Red = *vaccinia virus*–infected cells, Gray = non-loaded BV2 Microglia cells).

Over time, untreated SH-SY5Y and U87 spheroids continued to grow (Figure 6 and Figure 7). In spheroids directly treated with VV, visible infection was evident by 96 h post-treatment. When non-infected BV2 microglia were co-cultured with spheroids, the spheroid core showed increased green fluorescence over 96 h, indicating robust tumor cell proliferation.

In contrast, co-culture with VV-infected BV2 cells resulted in a gradual increase in red fluorescence, signifying successful viral transfer from microglia to cancer cells and subsequent infection. Fluorescence microscopy of sectioned spheroids revealed substantial structural disruption in models treated directly with VV or indirectly via VV-infected microglia, with reduced GFP signal and loss of organized 3D architecture, suggesting widespread cell death. Spheroids co-cultured with non-infected microglia maintained structural integrity and strong green fluorescence, consistent with the presence of viable cancer cells.

These findings indicate that both direct VV treatment and microglia-mediated viral delivery can effectively compromise tumor spheroid structure and viability.

## 4. Discussion

For the first time, our present study demonstrates that microglial cells infected with the VV strain LIVP 1.1.1 can effectively deliver the virus to neuroblastoma and glioblastoma cells in both 2D monolayers and 3D tumor spheroid models. VV-loaded microglia showed significant cytotoxicity, demonstrating enhanced efficacy in 3D spheroids, which suggests better viral infiltration and oncolytic distribution within tissue-like structures [13,14].

In pediatric oncology, oncolytic virotherapy offers a promising alternative or adjunct anticancer therapy. Celyvir, Pexa-Vec (JX-594), and Seneca Valley Virus (NTX-010) have demonstrated promising clinical resultsJX-594 has genetic alterations such as thymidine kinase gene deletion for tumor-selective replication, GM-CSF insertion for immunological stimulation, and β-galactosidase reporter [15,16,17,18]. A pediatric phase I trial found that JX-594 was well tolerated with moderate side effects and no dose-limiting toxicities. Four pediatric patients achieved stable illness [19]. In our own experiments, VV significantly inhibited SH-SY5Y neuroblastoma cell growth within 72–96 h.

In a study by Marcin Komorowski and colleagues, expression of C-X-C motif chemokine receptor 4 (CXCR4) antagonists from an oncolytic *vaccinia virus* significantly enhanced the therapeutic efficacy of dendritic cell (DC) vaccines compared to either a soluble CXCR4 antagonist or oncolysis alone, when administered intravenously to mice bearing neuroblastoma tumors. This work represents the first demonstration that modifying the tumor microenvironment with an armed oncolytic virus can markedly improve the performance of DC vaccines, resulting in robust and durable protection against neuroblastoma challenge [20].

In previous studies, VV-GD2m-NAP was produced by inserting NAP and GD2m genes into the Western Reserve (WR) strain. VV-GD2m-NAP decreased tumor growth and prolonged mouse survival in subcutaneous NSX2 neuroblastoma xenografts. Seneca Valley Virus NTX-010 (NCT01048892) was safe and tolerable in paediatric neuroblastoma patients in a phase I trial, with no dose-limiting toxicities [21].

GBM patients have an 8-month median survival rate despite decades of research and many treatment trials. GBM, the most frequent and aggressive malignant primary brain tumor, requires novel treatments. Oncolytic viral treatments are promising novel techniques. Oncolysis or modified viral vectors to deliver therapeutic genes directly to tumors are used in these methods [22].

Yijie Sun et al. [23] found that a next-generation VV designed to express IL-21 could treat mice with GL261 gliomas. The virus, given intratumorally or intravenously, changed the tumor microenvironment to generate a strong anti-tumor immune response, causing tumor reduction and longer survival in subcutaneous and orthotopic glioma models. Combining oncolytic VV with systemic α-PD1 immune checkpoint inhibition, which was unsuccessful alone, resulted in a synergistic effect that significantly improved treatment efficacy. Viral treatment expanded stem cell–like memory T cells, boosting anti-tumor immunity [23].

The cytotoxic effects of chemotherapy on immortalized and patient-derived glioma cells sensitive to VV-GMCSF-Lact were examined by Natalia Vasileva and colleagues. This virus was tested with temozolomide, the usual glioma treatment. Before viral infection, temozolomide had better anti-tumor effects than the reverse sequence. Both tumor growth inhibition and histological analysis showed that this sequential method was more effective in U87 MG glioblastoma xenograft models. VV-GMCSF-Lact monotherapy suppressed tumors better than temozolomide [24,25].

In our study, we found that VV can significantly inhibit U87 cell growth within 48 h, underscoring its potential for rapid therapeutic impact.

Despite promising preclinical results, clinical translation of OVs has been limited. OV oncolysis, viral tropism, delivery platforms, viral propagation, dosing methods, antiviral immunity, and to reach solid tumors, the OV must overcome many obstacles. External barriers prevent virus delivery since they have to pass through the endothelium to reach target cells. Solid tumours’ abnormal lymphatic networks, vascular hyperpermeability, and dense extracellular matrix (ECM) induce interstitial hypertension, which may limit viral penetration. OVs can potentially trigger a robust innate immune response because of interactions with APCs, broad antiviral immunity, preexisting antibodies, and blood factors like coagulation factors FIX, FX, and complement protein C4BP. OVs are more likely to be destroyed by the host’s immune system, making tumor invasion difficult [14]. Because of these limitations we used Microglia cells as a carrier for VV to enhance our virotherapy.

Microglia are strong candidates for therapeutic delivery due to their inherent tumor tropism, rapid motility, and ability to infiltrate dense neural tissue [26].

An in vitro study by Hwang et al. demonstrated that activated or engineered microglia can exert antitumor effects through the release of cathepsins, nitric oxide, and pro-inflammatory cytokines, or via delivery of therapeutic proteins such as IL-15 [17,20,27].

In the present study, we applied a novel carrier concept using BV2 cells loaded with oncolytic *vaccinia virus*. Our findings show that VV-infected BV2 cells significantly inhibited U87 glioblastoma cell growth, whereas non-infected BV2 cells had no measurable effect. These results support further investigation of microglia as delivery platforms for oncolytic viruses and other therapeutic agents targeting brain tumors.

Finally, Jee-Wei Emily Chen et al. studied patient-derived GBM cell-microglia interactions using brain-mimetic hydrogel. In 3D culture, microglial paracrine signaling significantly reduced GBM invasion by permitting secretome-based signalling and limiting cell interaction [28]. Consistent with these observations, our 3D spheroid experiments demonstrated that co-culturing VV-infected BV2 cells with U87 spheroids resulted in efficient viral transfer, widespread infection, and structural disruption of the cancer model. In contrast, spheroids co-cultured with non-infected BV2 cells retained green fluorescence indicative of viable cancer cells, despite some structural compromise.

To date, no studies have directly employed microglia as a deliberate therapeutic strategy to eliminate neuroblastoma cells. However, research examining the interactions between activated microglia and neuroblastoma or other neuronal tumor cells provides valuable insights into their potential anti-tumor functions.

The primary advantage of using microglia as cellular carriers rather than delivering free VV directly is their natural tumor-homing capacity, enabling them to migrate toward and infiltrate regions of the tumor that free virus may not efficiently reach, particularly in highly infiltrative tumors such as glioblastoma. Microglia can also shield the virus from neutralizing antibodies and other systemic immune barriers, potentially improving viral delivery and retention within the tumor microenvironment. Additionally, carrier cells may facilitate deeper penetration into dense 3D tumor structures, as demonstrated in our spheroid models.

We agree that this approach also presents clinical limitations, including the need to control viral replication within carrier cells, ensure safety regarding neuroinflammation or off-target infection, and address variability between murine BV2 microglia and human microglia in future translational studies. These points have now been clarified in the revised manuscript.

Moreover, tracking microglial migration, viral release dynamics, and the extent of bystander infection will be essential to determine whether this strategy improves intratumoral distribution, reduces off-target effects, and enhances synergy with radiotherapy or immunotherapy.

Using VV-infected microglia in vivo is particularly interesting for treating solid brain tumors, since delivering the virus is a challenge. In future in vivo experiments, VV-loaded microglia might be injected into brain tumor models using different methods. It could be introduced into the cerebrospinal fluid using intrathecal, intraventricular, or spinal channels. This would take advantage of their natural ability to move along CSF pathways [29,30].

### Study Limitations

Our study has the following limitations: first, all experiments were performed in vitro, and even though 3D spheroids better mimic tumor architecture, they cannot fully reproduce the complexity of in vivo brain tumors, including vascular, immune, and extracellular matrix interactions. Second, we used BV2 murine microglia, which may not fully represent the behavior of primary human microglia, potentially limiting translational relevance. Third, and as an important limitation of this study, is that cytokine and chemokine levels in the culture supernatants were not measured. Because microglia are highly responsive immunological cells, VV infection is likely to alter their inflammatory signaling profile, which could influence both viral replication dynamics and tumor–microglia interactions. Evaluating cytokines such as TNF-α, IL-6, IL-1β, and anti-inflammatory mediators would provide valuable insight into the activation state of VV-infected microglia and help clarify whether microglial secreted factors contribute to the observed cytotoxic effects. Although cytokine analysis was beyond the scope of the present work, future studies will incorporate comprehensive profiling of supernatants to better define the immunological consequences of microglial infection and to inform the design of subsequent in vivo experiments. Finally, the study did not evaluate safety considerations, such as neuroinflammation, off-target infection, or the impact of host immune responses, all of which will need to be addressed in our future in vivo work.

## 5. Conclusions

In the present study, we evaluated the therapeutic potential of microglial cells infected with the oncolytic *vaccinia virus* LIVP 1.1.1 against neuroblastoma and glioblastoma cells in both two-dimensional and three-dimensional in vitroculture systems. Our results demonstrate that infected microglia can efficiently deliver the virus to tumor cells, inducing substantial cytotoxic effects in both models. Notably, the 3D cultures, which more closely mimic the native tumor microenvironment, exhibited the most pronounced enhancement in oncolysis.

These results introduce a novel therapeutic paradigm that integrates cell-based delivery with oncolytic virotherapy, enabling targeted viral transfer to tumor cells via microglia. By leveraging the natural tumor-homing properties of microglia, this approach has the potential to overcome current barriers in treating aggressive brain and pediatric tumors, including poor viral penetration and immune evasion. While these findings are based on in-vitro models, their implications for precision-targeted cancer therapy are substantial. Future in vivo studies will be critical to validate efficacy, optimize delivery strategies, and assess safety for potential clinical translation.

Nevertheless, this study has several limitations. As mentioned above, our findings are based on in-vitro models and lack in vivo validation in animal models. We also used murine BV2 microglia together with a human tumor cell line (U87 and SH-SY5Y), which may not fully recapitulate the behavior of patient-derived microglia. Our work focused on phenotypic outcomes (viral spread, cytotoxicity, and spheroid disruption) and did not investigate the signaling pathways in VV-infected microglia and tumor cells. The specific antiviral, inflammatory, and cell death pathways altered by VV and microglia–tumor interactions remain unknown since we did not perform proteomics or other global molecular investigations.

## Data Availability

The datasets used and/or analyzed during the current study are available from the corresponding author on reasonable request.

## Abbreviations

The following abbreviations are used in this manuscript:

ANOVA	Analysis of Variance
ATCC	American Type Culture Collection
CAR	Chimeric Antigen Receptor
CNS	Central Nervous System
CV-1	African Green Monkey Kidney Fibroblasts (cell line)
CXCR4	C-X-C Motif Chemokine Receptor 4
DC	Dendritic Cell
DMEM	Dulbecco’s Modified Eagle Medium
EV	Extracellular Virus
FBS	Fetal Bovine Serum
GBM	Glioblastoma
GFP	Green Fluorescent Protein
GM-CSF	Granulocyte-Macrophage Colony-Stimulating Factor
IFNγ	Interferon Gamma
IL-15/IL-21	Interleukin-15/Interleukin-21
LIVP	Lister Institute of Viral Preparations (Vaccinia virus strain)
LPS	Lipopolysaccharide
MMP	Matrix Metalloprotease
MOI	Multiplicity of Infection
MTT	3-(4,5-dimethylthiazol-2-yl)-2,5-diphenyltetrazolium bromide
nAbs	Neutralizing Antibodies
NAP	Neutrophil-Activating Protein
OCT	Optimal Cutting Temperature (compound)
OV	Oncolytic Virus
PBS	Phosphate-Buffered Saline
PFU	Plaque-Forming Unit
poly(I:C)	Polyinosinic-polycytidylic acid
rAAV2	Recombinant Adeno-Associated Virus serotype 2
SEM	Standard Error of the Mean
TLRTLR9	Toll-like Receptor 9
VV	Vaccinia Virus
2D	Two-Dimensional
3D	Three-Dimensional
